# Supplementary material for: Real-world treatment trends and triple class exposed status in newly diagnosed multiple myeloma patients in Japan: A retrospective claims database study
Source: PLoS One. 2024 Sep 30;19(9):e0310333. doi: 10.1371/journal.pone.0310333 (PMC11441696; doi:10.1371/journal.pone.0310333)
Supplement: S1 Table — (DOCX) [file pone.0310333.s001.docx]

**S1 Table. Comorbidities defined using the ICD10 classification in this study**

| **Comorbidities** | **ICD10 code** | **ICD10 name of disease** |
| --- | --- | --- |
| Renal dysfunction | N00-N08 | Glomerular diseases |
|  | N10-N16 | Renal tubulo-interstitial diseases |
|  | N17-N19 | Renal failure |
|  | N25-N29 | Other disorders of kidney and ureter |
| Liver dysfunction | K70-K77 | Diseases of liver |
| Cardiac dysfunction | I05-I09 | Chronic rheumatic heart diseases |
|  | I11 | Hypertensive heart disease |
|  | I20-I25 | Ischaemic heart diseases |
|  | I26-I28 | Pulmonary heart disease and diseases of pulmonary circulation |
|  | I30-I52 | Other forms of heart disease |
| Pulmonary dysfunction | J60-J70 | Lung diseases due to external agents |
|  | J80-J84 | Other respiratory diseases principally affecting the interstitium |
|  | J95-J99 | Other diseases of the respiratory system |
| Vascular disorder | I10 | Essential (primary) hypertension |
|  | I60-I69 | Cerebrovascular diseases |
|  | I70-I79 | Diseases of arteries, arterioles and capillaries |
|  | I80-I89 | Diseases of veins, lymphatic vessels and lymph nodes, not elsewhere classified |
|  | I95-I99 | Other diseases of the respiratory system |
| Back pain | M54 | Dorsalgia |
| Dementia | F00-09 | Organic, including symptomatic, mental disorders |
