# Supplementary material for: Real-world treatment trends and triple class exposed status in newly diagnosed multiple myeloma patients in Japan: A retrospective claims database study
Source: PLoS One. 2024 Sep 30;19(9):e0310333. doi: 10.1371/journal.pone.0310333 (PMC11441696; doi:10.1371/journal.pone.0310333)
Supplement: S2 Table — (DOCX) [file pone.0310333.s002.docx]

**S2 Table.** **Proportion of patients per treatment regimen by age in 1^st^ line in the non-transplant group**

| **Treatment regimen** | **Total** | **Age** | | | |
| --- | --- | --- | --- | --- | --- |
|  |  | **<65 years** | **65-74 years** | **75-84 years** | **≥85 years** |
|  |  | **n (%)** | **n (%)** | **n (%)** | **n (%)** |
|  |  | **178** | **561** | **723** | **194** |
| Rd-based | 409 | 23 (12.9) | 98 (17.5) | 204 (28.2) | 84 (43.3) |
| Vd-based | 394 | 47 (26.4) | 138 (24.6) | 164 (22.7) | 45 (23.2) |
| RVd-based | 259 | 57 (32.0) | 109 (19.4) | 82 (11.3) | 11 (5.7) |
| DRd-based | 110 | 5 (2.8) | 41 (7.3) | 58 (8.0) | 6 (3.1) |
| Bor-based | 65 | 13 (7.3) | 26 (4.6) | 23 (3.2) | 3 (1.5) |
| DVd-based | 49 | 9 (5.1) | 21 (3.7) | 15 (2.1) | 4 (2.1) |
| VMP-based | 47 | 0 (0.0) | 18 (3.2) | 26 (3.6) | 3 (1.5) |
| Len-based | 43 | 1 (0.6) | 16 (2.9) | 18 (2.5) | 8 (4.1) |
| IRd-based | 13 | 0 (0.0) | 1 (0.2) | 12 (1.7) | 0 (0.0) |
| KRd-based | 4 | 2 (1.1) | 2 (0.4) | 0 (0.0) | 0 (0.0) |
| ERd-based | 4 | 0 (0.0) | 0 (0.0) | 4 (0.6) | 0 (0.0) |
| D-VMP-based | 4 | 0 (0.0) | 1 (0.2) | 3 (0.4) | 0 (0.0) |
| PVd-based | 2 | 1 (0.6) | 0 (0.0) | 0 (0.0) | 1 (0.5) |
| Dara-based | 2 | 0 (0.0) | 1 (0.2) | 1 (0.1) | 0 (0.0) |
| DKd-based | 1 | 1 (0.6) | 0 (0.0) | 0 (0.0) | 0 (0.0) |
| Other-based | 250 | 19 (10.7) | 89 (15.9) | 113 (15.6) | 29 (14.9) |
