# Supplementary material for: Real-world treatment trends and triple class exposed status in newly diagnosed multiple myeloma patients in Japan: A retrospective claims database study
Source: PLoS One. 2024 Sep 30;19(9):e0310333. doi: 10.1371/journal.pone.0310333 (PMC11441696; doi:10.1371/journal.pone.0310333)
Supplement: S3 Table — (DOCX) [file pone.0310333.s003.docx]

**S3 Table.** **Proportion of patients per treatment regimen by comorbidities in 1^st^ line in the non-transplant group**

| **Treatment regimen** | **Total** | **Comorbidities** | | | | |
| --- | --- | --- | --- | --- | --- | --- |
|  |  | **Renal dysfunction** | **Liver dysfunction** | **Cardiac dysfunction** | **Pulmonary dysfunction** | **Vascular disorder** |
|  |  | **n (%)** | **n (%)** | **n (%)** | **n (%)** | **n (%)** |
|  |  | **269** | **112** | **402** | **64** | **539** |
| Rd-based | 409 | 68 (25.3) | 26 (23.2) | 116 (28.9) | 20 (31.3) | 140 (26.0) |
| Vd-based | 394 | 88 (32.7) | 26 (23.2) | 115 (28.6) | 15 (23.4) | 155 (28.8) |
| RVd-based | 259 | 21 (7.8) | 19 (17.0) | 39 (9.7) | 10 (15.6) | 48 (8.9) |
| DRd-based | 110 | 17 (6.3) | 9 (8.0) | 24 (6.0) | 1 (1.6) | 35 (6.5) |
| Bor-based | 65 | 17 (6.3) | 7 (6.3) | 21 (5.2) | 3 (4.7) | 21 (3.9) |
| DVd-based | 49 | 10 (3.7) | 3 (2.7) | 14 (3.5) | 1 (1.6) | 16 (3.0) |
| VMP-based | 47 | 6 (2.2) | 3 (2.7) | 6 (1.5) | 3 (4.7) | 13 (2.4) |
| Len-based | 43 | 4 (1.5) | 1 (0.9) | 12 (3.0) | 1 (1.6) | 19 (3.5) |
| IRd-based | 13 | 1 (0.4) | 0 (0.0) | 4 (1.0) | 1 (1.6) | 2 (0.4) |
| KRd-based | 4 | 0 (0.0) | 1 (0.9) | 0 (0.0) | 0 (0.0) | 2 (0.4) |
| ERd-based | 4 | 0 (0.0) | 0 (0.0) | 1 (0.2) | 0 (0.0) | 1 (0.2) |
| D-VMP-based | 4 | 0 (0.0) | 0 (0.0) | 0 (0.0) | 0 (0.0) | 0 (0.0) |
| PVd-based | 2 | 0 (0.0) | 0 (0.0) | 1 (0.2) | 0 (0.0) | 0 (0.0) |
| Dara-based | 2 | 0 (0.0) | 0 (0.0) | 0 (0.0) | 0 (0.0) | 0 (0.0) |
| DKd-based | 1 | 0 (0.0) | 0 (0.0) | 0 (0.0) | 0 (0.0) | 0 (0.0) |
| Other-based | 250 | 37 (13.8) | 17 (15.2) | 49 (12.2) | 9 (14.1) | 87 (16.1) |
