# Supplementary figures and images for: Real-world treatment trends and triple class exposed status in newly diagnosed multiple myeloma patients in Japan: A retrospective claims database study
Source: PLoS One. 2024 Sep 30;19(9):e0310333. doi: 10.1371/journal.pone.0310333 (PMC11441696; doi:10.1371/journal.pone.0310333)

**S1 Fig. Study design**

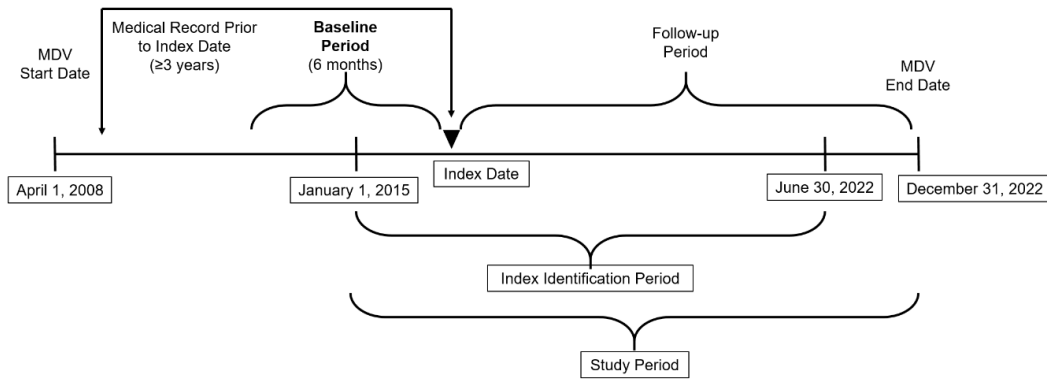

MDV, Medical Data Vision

Supplement: S1 Fig — MDV, Medical Data Vision. (PDF) [file pone.0310333.s004.pdf]
