# Supplementary material for: Real-world treatment trends and triple class exposed status in newly diagnosed multiple myeloma patients in Japan: A retrospective claims database study
Source: PLoS One. 2024 Sep 30;19(9):e0310333. doi: 10.1371/journal.pone.0310333 (PMC11441696; doi:10.1371/journal.pone.0310333)
Supplement: S2 Fig — (PDF) [file pone.0310333.s005.pdf]

S2 Fig. Definitions of treatment lines and line transfer for non-transplant group

Case 1: The case where the concomitant drugs in the regimens on the subsequent (next) treatment line did not include the drugs in the regimens on the current treatment line:

- If the initial prescription date in the subsequent MM therapy was within 28 days from the end date of the current treatment line, the treatment line was considered as not changed (Figure S2.1.1).

Figure S2.1.1

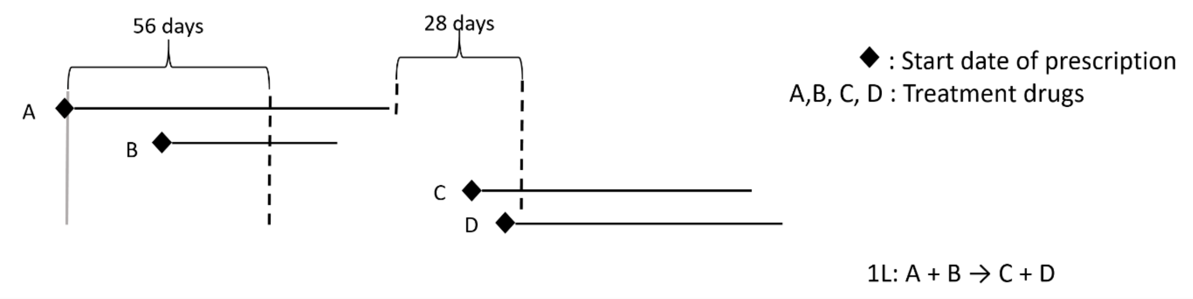

1L, 1st treatment line

- If the initial prescription date in the subsequent MM therapy was over 28 days from the end date of the current treatment line, the treatment line was considered as changed (Figure S2.1.2).

Figure S2.1.2

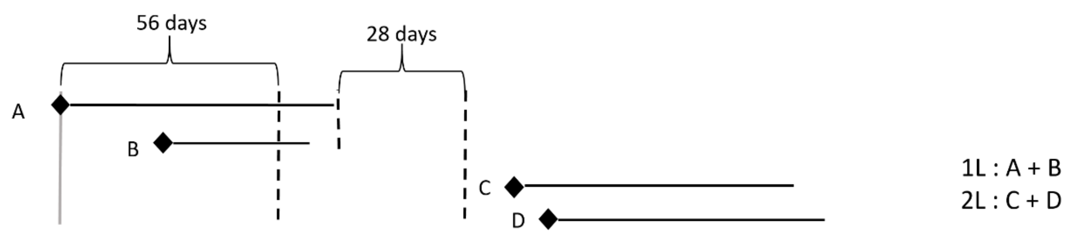

1L, 1st treatment line; 2L, 2nd treatment line

Case 2: The case where the concomitant drugs in the regimens on the subsequent (next) treatment line are the same drugs in the regimens of the current treatment line.

- If the initial prescription date in the subsequent MM therapy was within 90 days from the end date of the current treatment line, the treatment line was considered as not changed. (Figure S2.2.1).

Figure S2.2.1

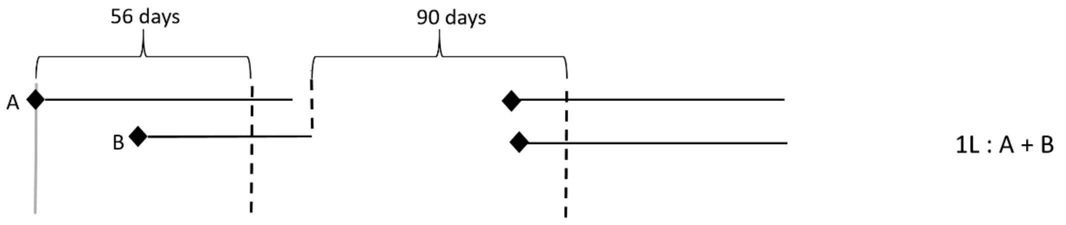

1L, 1st treatment line

- If the initial prescription date in the subsequent MM therapy was over 90 days from the end date of the current treatment line, the treatment line was considered as changed (Figure S2.2.2).

Figure S2.2.2

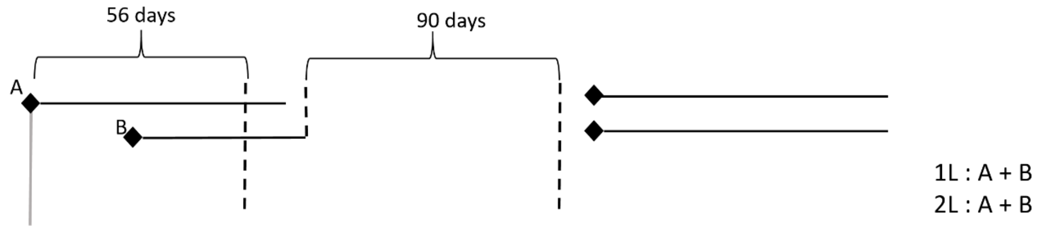

1L, 1st treatment line; 2L, 2nd treatment line

Case 3: When the regimens in the subsequent MM therapy contained a new drug into the regimens on the current treatment line.

- If the new drug was prescribed within the first 56-day period after the initial prescription date on the current treatment line, the treatment line was considered as not changed (Figure S2.3.1).

Figure S2.3.1

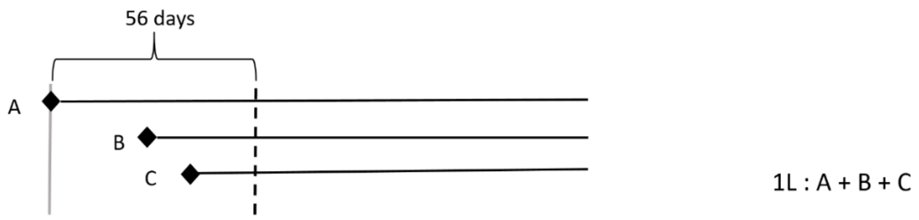

1L, 1st treatment line

- If the new drug was prescribed after the first 56-day period after the initial prescription date on the current treatment line and the new drug was prescribed within 28 days from the end date of the current treatment line, the treatment line was considered as not changed. (Figure S2.3.2)

Figure S2.3.2

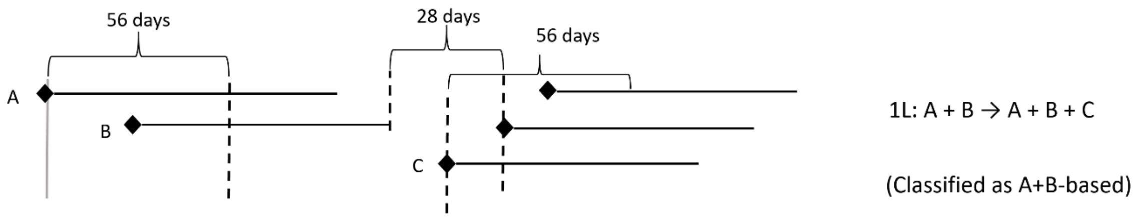

1L, 1st treatment line

- If the new drug was prescribed after the first 56-day period after the initial prescription date on the current treatment line and the new drug was prescribed after 28 days from the end date of the current treatment line, the treatment line was considered as changed. (Figure S2.3.3)

Figure S2.3.3

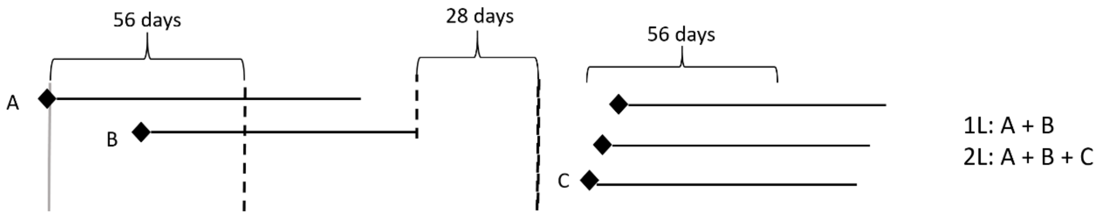

1L, 1st treatment line; 2L, 2nd treatment line

Case 4: When a part of the drugs in the regimens on the current treatment line was discontinued.

- If the initial prescription date in the subsequent MM therapy was within 28 days from the end date of the current treatment line, the treatment line was considered as not changed. (Figure S2.4.1).

Figure S2.4.1

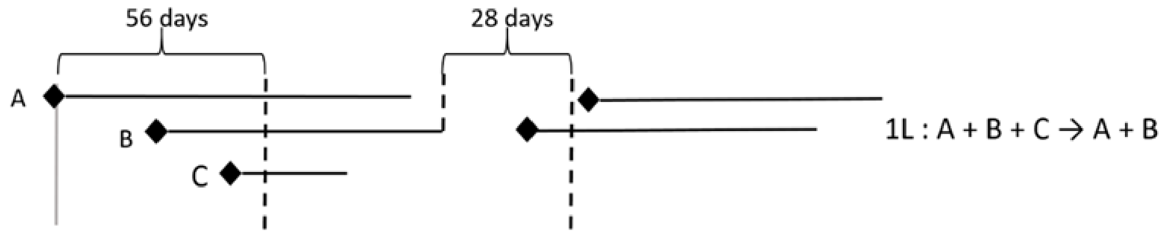

1L, 1st treatment line

- If the initial prescription date in the subsequent MM therapy was after 28 days from the end date of the current treatment line, the treatment line was considered as changed. (Figure S2.4.2).

Figure S2.4.2

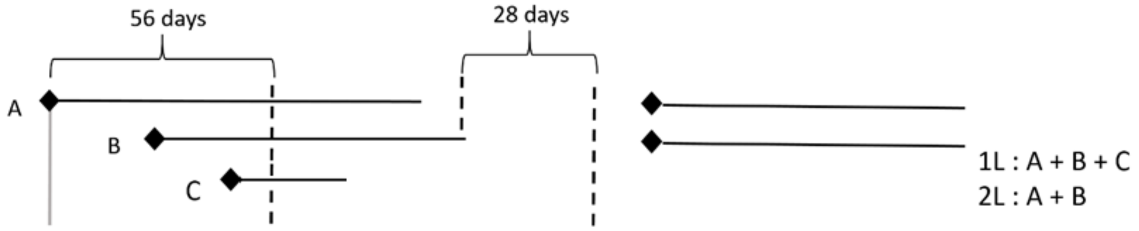

1L, 1st treatment line; 2L, 2nd treatment line

Case 5: When a part of the drugs in the regimens on the current treatment line was changed.

- If the initial prescription date in the subsequent MM therapy was within 28 days from the end date of the current treatment line, the treatment line was considered as not changed. (Figure S2.5.1)

Figure S2.5.1

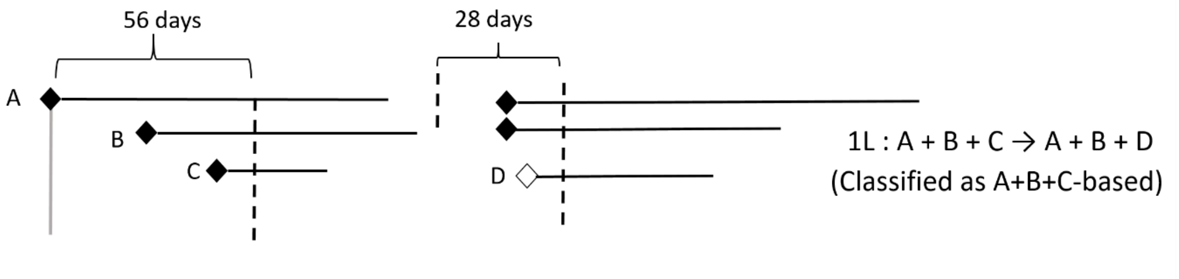

1L, 1st treatment line

- If the initial prescription date in the subsequent MM therapy was over 28 days from the end date of the current treatment line, the treatment line was considered as changed. (Figure S2.5.2)

Figure S2.5.2

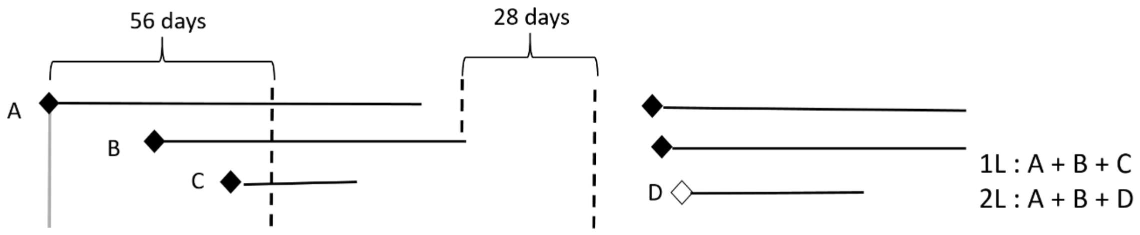

1L, 1st treatment line; 2L, 2nd treatment line

- If a part of drugs in the current MM therapy was changed after the end date of the first 56-day period after the initial prescription date of the current treatment line and the other drugs were continued, the treatment line was considered as not changed. (Figure S2.5.3)

Figure S2.5.3

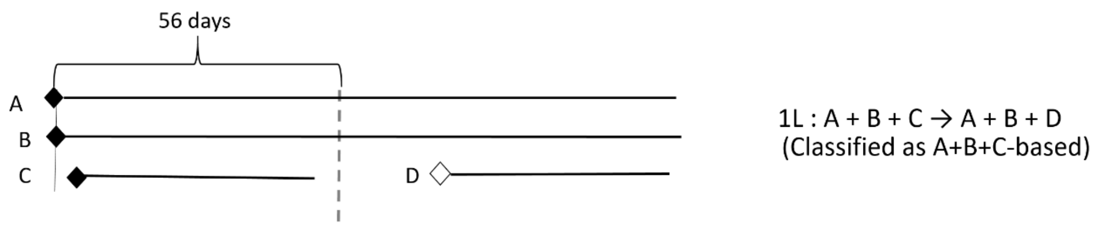

1L, 1st treatment line
