# Supplementary material for: Real-world treatment trends and triple class exposed status in newly diagnosed multiple myeloma patients in Japan: A retrospective claims database study
Source: PLoS One. 2024 Sep 30;19(9):e0310333. doi: 10.1371/journal.pone.0310333 (PMC11441696; doi:10.1371/journal.pone.0310333)
Supplement: S3 Fig — (PDF) [file pone.0310333.s006.pdf]

S3 Fig. Definitions of treatment lines and line transfer for transplant group.

Treatment regimens initiated within 120 days from transplantation were considered consolidation (in case the regimen included two drugs or over except melphalan, dexamethasone, and prednisolone) or maintenance therapy (in case the regimen included only one drug except melphalan, dexamethasone and prednisolone).

- If the treatment regimens after transplantation were defined as consolidation or maintenance therapy, the treatment line after the transplantation was the same line as before the transplantation. The maintenance regimens initiated within 28 days after consolidation therapy were considered the same line as before the transplantation. (Figure S3.1)

Figure S3.1:

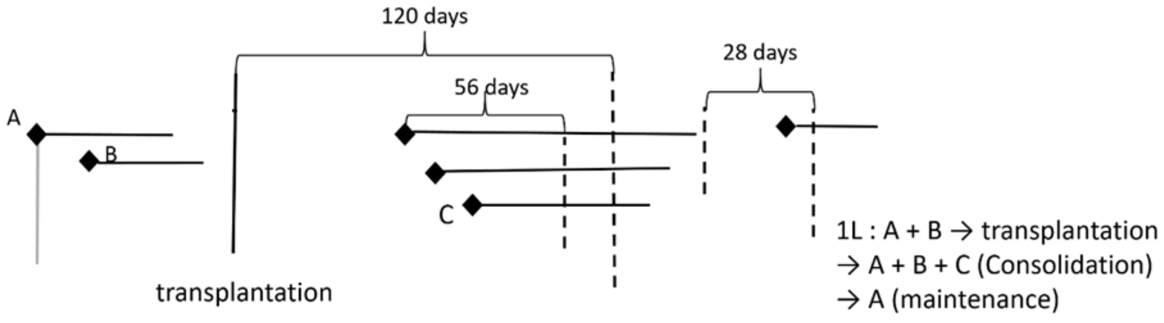

1L, 1st treatment line

- If the treatment regimens after transplantation are not defined as consolidation or maintenance therapy, the treatment line after the transplantation is considered to have been changed. (Figure S3.2)

Figure S3.2

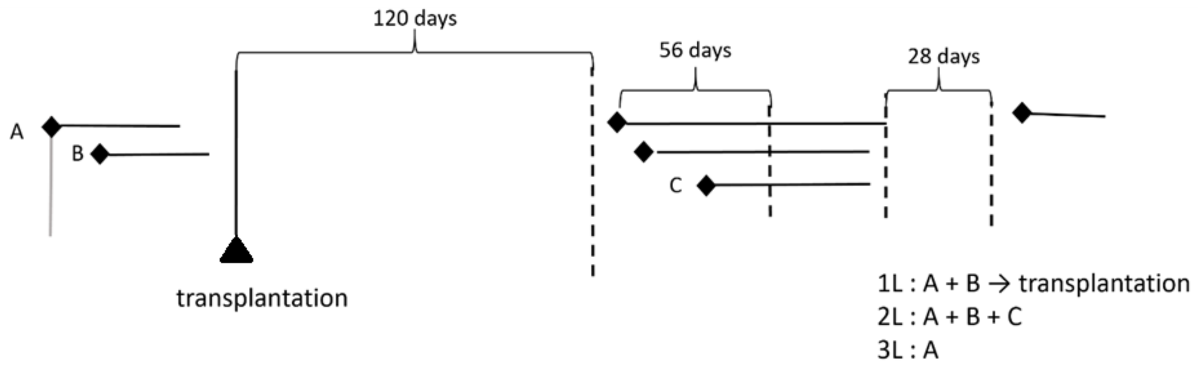

1L, 1st treatment line; 2L, 2nd treatment line; 3L, 3rd treatment line
