# Supplementary material for: Real-world treatment trends and triple class exposed status in newly diagnosed multiple myeloma patients in Japan: A retrospective claims database study
Source: PLoS One. 2024 Sep 30;19(9):e0310333. doi: 10.1371/journal.pone.0310333 (PMC11441696; doi:10.1371/journal.pone.0310333)
Supplement: Attachment — Submitted filename: Res to Reviewers comment_9Aug2024_clean.docx [file pone.0310333.s007.docx]

**Reviewers' comments:**

**Reviewer's Responses to Questions**

Comments to the Author

Thank you for your kind overall evaluation and your valuable time spent on reviewing our manuscript. We have answered your comments below and revised the manuscript accordingly. In addition, we have also revised according to comments from reviewers and made some corrections based on fact-check.

**1. Is the manuscript technically sound, and do the data support the conclusions?**

The manuscript must describe a technically sound piece of scientific research with data that supports the conclusions. Experiments must have been conducted rigorously, with appropriate controls, replication, and sample sizes. The conclusions must be drawn appropriately based on the data presented.

Reviewer #1: Partly

Reviewer #2: Yes

**Response:**

We have made minor revisions of the conclusion based on the data presented in this study as below.

“Our study showed that in the real-world setting, transplantation can be performed in patients with fewer comorbidities, aged up to 73 years. The present study showed that most transplant patients were hospitalized in large hospitals. In both non-transplant and transplant patients receiving 1st-line treatment, most received an Rd-based or Vd-based doublet regimen, or an RVd-based triplet regimen. Most TCE patients had received DRd-based, RVd-based, or DVd-based regimens. The proportion of TCE patients showed an increasing trend after 2nd or later lines for both non-transplant and transplant patients; this was highest in the transplant group. The findings of this study will support the development of treatment strategies and policies for MM treatment in real-world clinical practice in Japan.” (Page 27 of the track file of the revised manuscript)

_______________________________________

**2. Has the statistical analysis been performed appropriately and rigorously?**

Reviewer #1: No

Reviewer #2: Yes

**Response:**

In this study the data were summarized descriptively. The patient characteristics, duration of treatment, treatment pattern, and other variables in transplanted and non-transplanted patients who are newly diagnosed with MM were evaluated descriptively with summary statistics. This set of analyses is intended mainly for descriptive purposes and to provide a basis for future studies. *P*-values were not calculated to investigate any prespecified set of hypotheses. Further, all data analysis was performed using statistical software SAS version 9.4 (SAS Institute, Cary, NC, USA). We have already included all these details in the statistical analysis section in the manuscript file. (Last paragraph of statistical analysis section on Page 9 of the track file of the revised manuscript)

________________________________________

**3. Have the authors made all data underlying the findings in their manuscript fully available?**

The PLOS Data policy requires authors to make all data underlying the findings described in their manuscript fully available without restriction, with rare exception (please refer to the Data Availability Statement in the manuscript PDF file). The data should be provided as part of the manuscript or its supporting information, or deposited to a public repository. For example, in addition to summary statistics, the data points behind means, medians and variance measures should be available. If there are restrictions on publicly sharing data—e.g. participant privacy or use of data from a third party—those must be specified.

Reviewer #1: Yes

Reviewer #2: Yes

**Response:**

Thank you for your review and confirmation.

________________________________________

**4. Is the manuscript presented in an intelligible fashion and written in standard English?**

PLOS ONE does not copyedit accepted manuscripts, so the language in submitted articles must be clear, correct, and unambiguous. Any typographical or grammatical errors should be corrected at revision, so please note any specific errors here.

Reviewer #1: No

Reviewer #2: Yes

**Response:**

We have carefully looked over the manuscript and made some corrections for the language and consistency as below. We have got the manuscript edited by a native English editor as well and editor has made editorial changes throughout the manuscript. These changes are shown as track changes in the track version of the revised manuscript file.

_______________________________________

**5. Review Comments to the Author**

Please use the space provided to explain your answers to the questions above. You may also include additional comments for the author, including concerns about dual publication, research ethics, or publication ethics. (Please upload your review as an attachment if it exceeds 20,000 characters)

**Reviewer #1:**

Article title: Real-world treatment trends and triple class exposed status in newly diagnosed multiple myeloma patients in Japan: a retrospective claims database study.

In this manuscript, Moribe et al. describe the current treatment trends for patients with NDMM in Japan. The study does a good job of describing the regimens used across lines of therapy in both transplant-eligible and transplant-ineligible patients. It also describes the treatment trends according to underlying characteristics and comorbidities, which are useful for clinicians at the time of treatment selection. They also describe the percentage of triple-class exposed (TCE) patients across treatment lines. My main concerns are outlined below.

**Response:**

We greatly appreciate your positive feedback. Moreover, we also thank you for your kind advice and review comments. Here we are providing a point-by-point response.

1) Title: does not match with the manuscript as a whole. The authors discuss NDMM and RRMM. Perhaps the authors should consider a more broad title like A retrospective study to define treatment patterns for patients in Japan with newly diagnosed and relapsed and refractory multiple myeloma.

**Response:**

Thank you for your suggestion. As you pointed out, the results include data for both NDMM and RRMM patients. However, this study mainly focused on NDMM patients rather than RRMM patients after strict definition of NDMM patients. Moreover, as for RRMM setting, the data has just been presented as the treatment trend and TCE status among NDMM patients receiving 2^nd^, 3^rd^, 4^th^, and 5^th^ or later lines of treatments. Therefore, we would like to keep the title unchanged.

2) Abstract: A bit all over the place.

• The authors need to comment as to why of the 1780 patients, why only 128 were considered transplant eligible. Why did they not include more transplant eligible patients?

• They majority of the abstract is focused on the NDMM patients and not RRMM.

• How is it that by the 5th line that only 21.2% and 43.8% of patients were triple class exposed?

**Response:**

• This is a limitation of this study. As Japanese patients with MM are very elderly (with median age >75 years) and have multiple comorbidities, only 20% of NDMM patients are eligible for transplantation. Furthermore, in this study, NDMM patients who had a follow-up period of ≥6 months from the index date have been selected. When the patients had shorter follow-up period, the medical record for transplantation might not be detected even if the patients have received transplantation after the follow-up period in this study. Therefore, the number of transplant eligible patients may be underestimated.

• As you pointed out, this study focused mainly on NDMM patients rather than RRMM patients.

• The NDMM patients have been first identified according to inclusion and exclusion criteria and then evaluated the TCE situation in each treatment line for the patients. However, because the follow-up period was ≥6 months from the index date, more than half of the NDMM patients remained in the 2^nd^ line. Just a few patients transferred up to the 5^th^ line. Thus, the MM patient number has gradually become less from the 1^st^ line to the 5^th^ line, though the TCE patient number has gradually increased from the 1^st^ line to the 5^th^ line. Therefore, the cumulative TCE patients in the 5^th^ line may be underestimated.

3) Line 70: This sentence does not make any sense and should be rewritten.

**Response:**

According to your comment, we have rewritten the sentence as below.

“However, daratumumab combination regimens used in the 1st line have complicated MM treatment and fragmented later-line MM treatment sequences.” (Last three lines of first paragraph on Page 4 of the track file of the revised manuscript)

4) Line 74: It is untrue that triple class exposed patients have limited treatment options. Almost all patients will have been triple class exposed by the end of 2nd line therapy (at the very latest) and most will be TCE during early induction. Are the authors considering TCE to be equivalent to triple class refractory (TCR). If so, this needs to be changed throughout the manuscript. Additionally, with the addition of CAR and bispecifics, treatment options are now considerably more than they were even a few years ago - this should be highlighted.

**Response:**

As you pointed out, most patients will have been TCE by the end of 2^nd^ line therapy most recently, since DRd and D-VMP regimens have been integrated in the 1^st^ line. However, because the study period was from January 1, 2015 to December 31, 2022, the most patients in this study should have been TCE in later lines than the 2^nd^ line.

We have added this information into the description as below:

“Our results suggested that there was an increasing trend in the proportion of TCE patients, especially in transplant group patients (cumulative TCE rate; 21.2% and 43.8% in the non-transplant and transplant groups, respectively). Since DRd and D-VMP regimens have been integrated into 1st-line treatment, most patients are now TCE by the end of 2nd-line therapy. However, because the study period was from January 1, 2015 to December 31, 2022, the most patients in the study should have been TCE in later lines than the 2nd line.” (Second paragraph on Page 25 of the track file of the revised manuscript)

Further, we consider TCE is not equivalent to TCR. Most patients with MM who are TCE will eventually have relapse and TCE patients who have relapsed or are refractory may have a poor prognosis with worsening outcomes and limited treatment options in clinical practice considering the physical condition of patients. Below are the references:

- Gandhi UH, Cornell RF, Lakshman A et al. Outcomes of patients with multiple myeloma refractory to CD38-targeted monoclonal antibody therapy. Leukemia 33(9), 2266–2275 (2019).
- Kumar SK, Dimopoulos MA, Kastritis E et al. Natural history of relapsed myeloma, refractory to immunomodulatory drugs and proteasome inhibitors: a multicenter IMWG study. Leukemia 31(11), 2443–2448 (2017).
- Nijhof IS, van De Donk N, Zweegman S, Lokhorst HM. Current and new therapeutic strategies for relapsed and refractory multiple myeloma: an update. Drugs 78(1), 19–37 (2018).
- Mikhael J. Treatment options for triple-class refractory multiple myeloma. Clin. Lymphoma Myeloma Leuk. 20(1), 1–7 (2020).

We agree with your comment “with the addition of CAR and bispecifics, treatment options are now considerably more than they were even a few years ago”. According to your comment, we revised the description as below.

“TCE patients have limited treatment options, especially before chimeric antigen receptor T-cell (CAR-T) therapies and bispecific antibodies (BsAbs) become available.” (Second paragraph on Page 4 of the track file of the revised manuscript)

5) Line 114: It is important to highlight the time period in which patients had data collected and not just when the data was captured. The results suggest that patients treated with an initial diagnosis before 2015 were included. This is unclear.

**Response:**

In this study, the results do not include the data for patients treated with an initial diagnosis before 2015 (before the study period), because we focused the time period after lenalidomide has become available for NDMM patients in Japan. To clearly define incident NDMM diagnosis in the study period in this study, we excluded patients who did not have any medical record for at least for 3 years or had received anti-myeloma treatments and MM diagnosis before the index date. Moreover, even if we changed the window period of 3 years (to 2 years, 1 year and 6 months), the patient characteristics did not change materially, suggesting minimal possibility of bias.

We added description to clarify this point as below.

“...Multiple myeloma and malignant plasma cell neoplasms), had ≥6 months follow-up data, and were prescribed bortezomib, lenalidomide, and/or daratumumab after the 1^st^ diagnosis of MM were included in the study. Therefore, the results did not include the data of treated patients with an initial diagnosis before 2015.” (First paragraph of ‘Study population and study period’ on Page 6 of the track file of the revised manuscript)

6) Line 119-120 – Should read “ Patients were excluded from the study if they were prescribed anti-myeloma treatment drugs other than dexamethasone and prednisone before index date”

Line 124 – The definition of index date should be moved up to before the concept is first utilized (page 6 line 120). To make it easier for the reader.

**Response:**

We agree with your suggestion. We moved the definition of index date up just after explaining the study period and inclusion criteria.

Therefore, the results do not include the data of treated patients with an initial diagnosis before 2015. The index date was defined as the earliest prescription date of the 1st-line drugs in the same month or months after the first diagnosis of MM. (First paragraph of ‘Study population and study period’ on Page 6 of the track file of the revised manuscript)

7) Line 150 – Authors mentioned that the number of transfusions was a variable of interest, but this was never addressed.

**Response:**

We apologize for this error. We deleted transfusion and corrected this description as follows:

“Specific outcome variable of interest was TCE status in each line.” (Paragraph above ’Statistical analysis’ on Page 8 of the track file of the revised manuscript)

8) Page 9; Study population – Although Figure 1 does a great job at explaining the breakdown of patients, the text is a little confusing and should be rewritten.

**Response:**

We have rewritten the text to explain the study population as below.

Over the study period, 44,897 patients had a confirmed diagnosis of MM, of which 16,021 patients had prescriptions for daratumumab, lenalidomide, and/or bortezomib at the index date (Fig 1). Of 12,255 patients who had a follow-up period of at least 6 months from the index date, 10,471 patients who met the other three exclusion criteria—were prescribed anti-myeloma treatment drugs other than dexamethasone and prednisone before the index date , had no medical records before the index date for ≥3 years, and received SCT before the index date—were excluded. Thus, 1,784 patients were included in the final cohort analysis set. Of the 1,784 patients, 1,656 patients were transplant ineligible (non-transplant group) and 128 patients were transplant eligible (transplant group) (Fig 1). (‘Study population’ paragraph on Page 10 of the track file of the revised manuscript)

9) Line 188: Again, this is a very skewed population. Is autoHSCT not standarly used in Japan?

**Response:**

This is a limitation of this study. As Japanese patients with MM are elderly (with median age >75 years) and have multiple comorbidities, around 20% of NDMM patients are eligible for transplantation. Furthermore, in this study, NDMM patients who had a follow-up period of at least 6 months from the index date have been selected. When the patients had shorter follow-up period, the medical record for transplantation might not be detected even if the patients have received transplantation. Therefore, the number of transplant eligible patients may be underestimated.

We have added this as limitation as below.

Second, in Japan, around 20% of NDMM patients are eligible for transplantation [5,22]. This study selected patients with NDMM with ≥6 months follow-up from the index date; therefore, patients with shorter follow-up would be excluded even if they were eligible for, or had received, transplantation, resulting in underestimation of the number of patients eligible for transplantation. (Limitations paragraph on Page 20 of the track file of the revised manuscript).

10) Line 198+: How are comorbidities defined. If this data is included, the authors need to explain what is meant by renal dysfunction, etc (e.g. CrCl between X and Y)

**Response:**

Comorbidities have been defined using the ICD10 classification (please see S1table below) as diseases whose diagnostic records existed within 6 months before the index date for each patient. We added description as follows:

“Comorbidities were defined using the ICD10 classification codes recorded within 6 months before the index date (S1 table).” (First paragraph of variables section on Page 6 of the track file of the revised manuscript)

Please also check the additional supplementary table (S1 table).

**S1 Table. Comorbidities defined using the ICD10 classification in this study**

| **Comorbidities** | **ICD10 code** | **ICD10 name of disease** |
| --- | --- | --- |
| Renal dysfunction | N00-N08 | Glomerular diseases |
|  | N10-N16 | Renal tubulo-interstitial diseases |
|  | N17-N19 | Renal failure |
|  | N25-N29 | Other disorders of kidney and ureter |
| Liver dysfunction | K70-K77 | Diseases of liver |
| Cardiac dysfunction | I05-I09 | Chronic rheumatic heart diseases |
|  | I11 | Hypertensive heart disease |
|  | I20-I25 | Ischaemic heart diseases |
|  | I26-I28 | Pulmonary heart disease and diseases of pulmonary circulation |
|  | I30-I52 | Other forms of heart disease |
| Pulmonary dysfunction | J60-J70 | Lung diseases due to external agents |
|  | J80-J84 | Other respiratory diseases principally affecting the interstitium |
|  | J95-J99 | Other diseases of the respiratory system |
| Vascular disorder | I10 | Essential (primary) hypertension |
|  | I60-I69 | Cerebrovascular diseases |
|  | I70-I79 | Diseases of arteries, arterioles and capillaries |
|  | I80-I89 | Diseases of veins, lymphatic vessels and lymph nodes, not elsewhere classified |
|  | I95-I99 | Other diseases of the respiratory system |
| Back pain | M54 | Dorsalgia |
| Dementia | F00-09 | Organic, including symptomatic, mental disorders |

11) Table 4 - It is not clear to me what the difference between the cumulative number and proportion of patients using therapies across different treatment lines and the number and proportion of patients based on the most recent treatment lines being used is, this needs further explanation.

**Response:**

Cumulative number and proportion of patients using therapies across different treatment lines” represents the total number of patients who have moved to each treatment line and experienced treatment in that line. “Number and proportion of patients based on the most recent treatment lines being used” represents the patients who are currently receiving treatment in each treatment line. We added this explanation as footnote into Table 4. (Page 14 below Table 4)

12) Table 5: This table is unclear and confusing. It is not clear how duration of treatment in 1st line treatment is broken down between patients prescribed and not prescribed subsequent treatment. Are you saying that duration of treatment to 1st line (non-transplant) in those patients treated with more than one line of therapy was 6.9 months vs 14.3 months in those patients that didn't get more than one line of treatment?

**Response**

Thank you for your comment.

When patients in 1^st^ line of treatment moved to 2^nd^ line of treatment, their follow-up time was broken down: treatment duration for 1^st^ line of treatment was terminated and subsequent period was counted as for 2^nd^ line of treatment (and so on). Let us take an example of a patient in non-transplant group who took total of 12 months of treatment, of which 6 months was in 1^st^ line, 4 months in 2^nd^ line, and 2 months in 3^rd^ line when he died. Then, he would contribute 6 months in 1^st^ line (Prescribed group), 4 months in 2^nd^ line (Prescribed group), and 2 months in 3^rd^ line (Not prescribed group).

So, we agree to your interpretation that median duration of treatment for 1^st^ line (non-transplant) in those patients treated with more than one line of therapy was 6.9 months vs 14.3 months in those patients who did not get more than one line of treatment.

We corrected label in Table 5 as below to add to clarity:

**Table 5. Duration of treatment in non-transplant and transplant groups.**

| **Treatment line** | **Duration of treatment (months)** | | | | | |
| --- | --- | --- | --- | --- | --- | --- |
|  | **Non-transplant group** | | | **Transplant group** | | |
|  | **Overall** | **Subsequent line treatment** | | **Overall** | **Subsequent line treatment** | |
|  |  | **Prescribed** | **Not prescribed** |  | **Prescribed** | **Not prescribed** |
|  | **n=1,656** | **n=605** | **n=1,051** | **n=128** | **n=76** | **n=52** |
| 1^st^ line | 10.86  (0.03–95.08) | 6.90  (0.03–70.67) | 14.32  (0.03–95.08) | 11.76  (0.43–76.06) | 4.80  (0.43–66.60) | 18.96  (4.70–76.06) |
| 2^nd^ line | 7.56  (0.03–82.46) | 6.46  (0.03–44.85) | 8.51  (0.03–82.46) | 8.08  (0.10–67.12) | 2.92  (0.69–56.21) | 9.91  (0.10–67.12) |
| 3^rd^ line | 4.48  (0.03–66.60) | 4.07  (0.07–40.67) | 4.76  (0.03–66.60) | 8.23  (0.46–47.77) | 4.01  (0.46–15.80) | 10.02  (1.97–47.77) |
| 4^th^ line | 5.29  (0.03–43.89) | 3.45  (0.23–15.61) | 7.00  (0.03–43.89) | 4.47  (0.10–30.55) | 3.25  (0.72–6.44) | 7.54  (0.10–30.55) |

Duration of treatment in months expressed as median (minimum–-maximum)

13) Table 6 - It would be more visually impactful if the table were reorganized to display the treatment regimens for the non-transplant and the transplant groups side by side rather than in sequential order, similar to Table 7.

**Response:**

Thank you for the suggestion., However, the data of table 6 are also presented in Figure 2 in the same order. So, we would like to retain the same order in Table 6 for consistency with Figure 2.

14) "Frequently used treatment regimen": The first paragraph in this section needs clarity. The data is not presented in a way that is useful or informative. The table helps clarify this a bit but the authors should consider rewriting this section so that it is more clear. Additionally, in Table 6, the authors include IRd - has ixazomib been introduced anywhere in the manuscript? What about F, Sar, S, etc.? Consider moving all of the regimens that are not discussed in the body of the manuscript to the supplemental section.

**Response:**

Thank you for your suggestion. Data of Table 6 are presented in Figure 2. We think that Table 6 should show all the regimens identified in this study to support good understanding of Figure 2. Also, please note that Table 1 in this manuscript provides details of all the drug regimens used.

We have revised the first paragraph according to your suggestion as below.

Of 1,656 non-transplant patients, 24.7% received lenalidomide and dexamethasone (Rd)-based 1st-line treatment, 23.8% received bortezomib and dexamethasone (Vd)-based, and 15.6% received lenalidomide, bortezomib, and dexamethasone (RVd)-based therapies: for 2nd-line treatment, the corresponding proportions were 22.0%, 9.8%, and 3.6%, respectively (Table 6 and Fig 2). Together, the Rd-, Vd-, or RVd-based therapies were used to treat 64.1%, 35.4%, 21.1%, and 13.3% patients in 1st-, 2nd-, 3rd-, and 4th-line treatments, respectively. (‘Frequently used treatment regimens paragraph’ on Pages 15 and 16 of the track file of the revised manuscript)

15) Line 244: 24.7, 23.8, and 15.6 don't add up to 100%. What else were patients treated with? I thought the inclusion was that patients had to be treated with len, bort, or a combination of these.

**Response:**

Thank you for this comment. We have commented on the most commonly used three regimen groups here: 24.7% , 23.8% and 15.6% patients received treatment with Rd-based, Vd-based and RVd-based regimens during the 1^st^ line treatment. The remaining 35.9% patients received other regimen treatments like Len-based, Bor-based or Dara-containing regimens (Please see Table 6 for details).

16) Line 247: Not sure this makes sense. 24.7% of patients received Rd in 1st line and then 22% received Rd in the 2nd line. It would be more useful to understand the treatment pattern in my opinion. For example, did physicians change from Rd to Vd or Rd to RVd?

**Response:**

Thank you for your comment. We analyzed the treatment regimen sequence pattern in non-transplant group. According to our internal data, 23.8% and 16.4% of patients received Rd-based in the 1^st^ line treatment moved to Rd-based and Vd-based in the 2^nd^ line treatment, respectively. However, because of limited patient population, we could not clearly show the treatment sequence patterns in the manuscript.

17) Line 261: Top 3? Perhaps use wording like the three more frequently prescribed.

**Response:**

Thank you for your suggestion. According to your comment, we have revised the description as below.

The three most frequently prescribed regimens for 1st-line induction therapy before SCT were RVd-based (49.5%), Vd-based (18.7%), and DVd-based (8.4%). For 2nd-line induction therapy, bortezomib-based (25.0%), Rd-based (20.0%), and carfilzomib, lenalidomide, and dexamethasone (KRd)-based or carfilzomib and dexamethasone (Kd)-based (each 10.0%) were prescribed most frequently (Table 6 and Fig 2). (First paragraph of Page 18 of the track file of the revised manuscript)

18) Line 281: "in the TCE situation" should be rewritten

**Response:**

Thank you for pointing out. We appropriately corrected as below.

in TCE situation. → in TCE group (Pages 19 and 20 of the track file of the revised manuscript)

19) Table 7: Again, I am confused how any patients in the 4th or 5th line could not be triple class exposed. Also, with the attrition rate being so high, is cumulative TCE meaningful?

**Response:**

The NDMM patients have been first identified according to inclusion and exclusion criteria and then evaluated for inclusion in the TCE group in each treatment line for the patients. However, because the follow-up period was ≥6 months from the index date, more than half of the NDMM patients remained in the 2^nd^ line. Just a few patients transferred up to the 5th line. Thus, the MM patient number gradually reduced from the 1st line to the 5th line, though the number of patients in TCE group gradually increased from the 1^st^ line to the 5^th^ line. Therefore, the cumulative TCE patients in the 5^th^ line may be underestimated.

Despite this limitation, the cumulative TCE data are meaningful allowing comparison with other studies in the future.

20) Line 294: This paragraph needs to be cleaned up, the description is not very clear though the information is important.

**Response:**

Thank you for your suggestion. According to your comment, we have revised the description as below.

The majority (89.7%) patients who were TCE in the 1st line had become so due to sequential therapies of multiple regimens, Vd-based to DRd-based sequencing being the most common (15.8%), followed by RVd-based to DRd-based (9.2%), and Rd-based to DVd-based (4.3%). One tenth (10.3%) of TCE patients had received a 1st-line four-drug combination regimen (daratumumab, lenalidomide, bortezomib, and dexamethasone). (First paragraph of Page 20 of the track file of the revised manuscript)

21) Line 315: Need to discuss why more transplant patients were not captured in this data set.

**Response:**

We have added the description below as second limitation into the text.

First, the exclusion criteria that were set to identify accurately patients with NDMM—especially excluding patients with no anti-myeloma treatment records for at least 3 years before the index date—may have excluded patients unnecessarily. Second, in Japan, around 20% of NDMM patients are eligible for transplantation [5,22]. This study selected patients with NDMM with ≥6 months follow-up from the index date; therefore, patients with shorter follow-up would be excluded even if they were eligible for, or had received, transplantation, resulting in underestimation of the number of patients eligible for transplantation. (Limitation part on Page 26 of the track file of the revised manuscript)

References:

5. Guan J, Tanaka S, Yamada S, Sato I, Kawakami K. Treatment patterns in newly diagnosed multiple myeloma patients in Japan using a large-scale claims database: retrospective cohort study. Japan J Pharmacoepidemiol/Yakuzai Ekigaku. 2020;25: 43-53. doi: 10.3820/jjpe.25.e2

22. Ozaki S, Handa H, Saitoh T, Murakami H, Itagaki M, Asaoku H. Trends of survival in patients with multiple myeloma in Japan: a multicenter retrospective collaborative study of the Japanese Society of Myeloma. Blood Cancer J 2015;5: e349. doi: 10.1038/bcj.2015.79

22) Lines 348-350 – The authors comment on utilizing K vs V in the second line mostly because of the difference in adverse events (namely peripheral neuropathy) – However, there should also be a mention of the difference in efficacy of K vs. V and also that as they clearly described nearly 50% of patients received bortezomib in the first line, making carfilzomib a better PI option in the second and further lines.

**Response:**

Thank you for your suggestion. According to your comment, we have revised this part as below.

For patients who received transplantation in the 2nd line, regimens containing carfilzomib (Kd or KRd) were preferentially selected versus those containing bortezomib, as these regimens are associated with higher response rates and lower incidence of peripheral neuropathy [14]. As this study showed that bortezomib or bortezomib-containing regimens (RVd, Vd, DVd) were used in 78.5% of transplant patients for 1st-line induction therapy (Table 6 and Fig 2), carfilzomib would be the preferable PI for 2nd-line induction therapy. (Pages 22 and 23 of the track file of the revised manuscript)

23) Line 357 onwards – The authors’ comment mentioned that “Lenalidomide containing regimens should be generally avoided because of their characteristic side effects, DVT and renal excretion pathway.” – This should be addressed as there is evidence that lenalidomide can be utilized in patients with renal dysfunction even in those undergoing HD as long as the dose is adjusted to the Creatinine Clearance. This statement might wrongly prevent providers from using lenalidomide, which, as we know, is a cornerstone of MM treatment in patients with any degree of renal dysfunction. Similarly, the authors also mentioned, “On the other hand, in patients with cardiac and pulmonary dysfunctions, Vd should be avoided because bortezomib was characterized by cardiac and pulmonary toxicities.” – In the ENDURANCE trial, only 5% of patients experienced at least one grade 2 or higher CV and pulmonary AE, and in most cases these were reversible. This statement could also wrongly lead clinicians to avoid bortezomib in patients with any degree of cardiopulmonary disease and should be modified.

**Response:**

We agree with your point.

Lenalidomide can be utilized in patients with renal dysfunction even in those undergoing HD, as long as the dose is adjusted according to the Creatinine Clearance. Bortezomib can be utilized in patients with cardiopulmonary disease even by dose adjustment and subcutaneous administration. Therefore, the statements might wrongly prevent physicians from using lenalidomide and bortezomib. According to your comment, we have revised the part as below, in an evidence-based manner.

For patients aged 65–75 years, a Vd-based regimen was preferred over an RVd triplet regimen. In patients with renal dysfunction, a Vd-based regimen was preferred because of its effectiveness in improving renal dysfunction and no requirement for dose modification, regardless of creatinine clearance levels [16–17]. Patients with hepatic dysfunction were prescribed Rd or Vd, rather than RVd: this was also the case for patients with cardiac and vascular dysfunction. In agreement with previously published studies, the present study suggests that treatment pattern and regimen choice (doublet vs. triplet) should be based on the drugs’ safety profiles, and patients’ overall physical condition and the presence of comorbidities [18]. (Second paragraph of Page 23 of the track file of the revised manuscript)

16. Dimopoulos MA, Roussou M, Gavriatopoulou M. Bortezomib-based triplets are associated with a high probability of dialysis independence and rapid renal recovery in newly diagnosed myeloma patients with severe renal failure or those requiring dialysis. Am J Hematol. 2016;91: 499-502. doi: 10.1002/ajh.24335

17. Dimopoulos MA, Merlini G, Bridoux F. Management of multiple myeloma-related renal impairment: recommendations from the International Myeloma Working Group. Lancet Oncol. 2023;24: e293-e311. doi: 10.1016/S1470-2045(23)00223-1

24) Discussion: Much too long, this needs to be shortened and written more concisely. A segment focusing on proposing the standardization of quadruplet therapy in NDMM should be added – if this aligns with the resources and available drug approval in Japan.

**Response:**

Thank you for your suggestion. According to your comment, we have revised and shortened the discussion section. We have made the discussion part concise by reducing approximately 250 words. (Revised discussion section from Page 20 to Page 27 of track file of the revised manuscript).

25) Conclusion: Sentences 1 and 2 in this paragraph are not the reason for this study and this data was not highlighted in the manuscript, so why do the authors include it here?

**Response:**

Thank you for your suggestion. According to your comment, we have revised the sentences 1 and 2 in the paragraph of conclusion based on the data presented in this study as below.

Conclusion

Our study showed that in the real-world setting, transplantation can be performed in patients with fewer comorbidities, aged up to 73 years. The present study showed that most transplant patients were hospitalized in large hospitals. In both non-transplant and transplant patients receiving 1st-line treatment, most received an Rd-based or Vd-based doublet regimen, or an RVd-based triplet regimen. Most TCE patients had received DRd-based, RVd-based, or DVd-based regimens. The proportion of TCE patients showed an increasing trend after 2nd or later lines for both non-transplant and transplant patients; this was highest in the transplant group. The findings of this study will support the development of treatment strategies and policies for MM treatment in real-world clinical practice in Japan. (Page 27 of the track file of the revised manuscript)

**Reviewer #2:**

Thanks to the authors for the article. A useful study reflecting real life data. The study nicely discussed the treatments applied to patients diagnosed with multiple myeloma in Japan. I am just wondering why there is a significant difference in the number of transplant-ineligible and transplant-eligible patients.

**Response:**

We deeply appreciate your positive feedback and your comment to the manuscript.

We understand your point. This is a limitation of this study. As Japanese patients with MM are elderly (with median age >75 years) and have multiple comorbidities, around 20% of NDMM patients are eligible for transplantation. Furthermore, in this study, NDMM patients who had a follow-up period of at least 6 months from the index date have been selected. When the patients had shorter follow-up period, the medical record for transplantation might not be detected even if the patients have received transplantation after the follow-up period in this study. Therefore, the number of transplant eligible patients may be underestimated.

We added the description below as second limitation into the text.

First, the exclusion criteria that were set to identify accurately patients with NDMM—especially excluding patients with no anti-myeloma treatment records for at least 3 years before the index date—may have excluded patients unnecessarily. Second, in Japan, around 20% of NDMM patients are eligible for transplantation [5,22]. This study selected patients with NDMM with ≥6 months follow-up from the index date; therefore, patients with shorter follow-up would be excluded even if they were eligible for, or had received, transplantation, resulting in underestimation of the number of patients eligible for transplantation. Additional limitations are common to database studies: the MDV database contains claims data, rather than comprehensive medical and treatment histories of all patients as this information is not captured prior to database registration or outside registered hospitals, and is censored if the patient changes hospital. (Limitation section on page 26 of the track file of the revised manuscript)

________________________________________

**6. PLOS authors have the option to publish the peer review history of their article (what does this mean?). If published, this will include your full peer review and any attached files.**

If you choose “no”, your identity will remain anonymous but your review may still be made public.

Do you want your identity to be public for this peer review? For information about this choice, including consent withdrawal, please see our Privacy Policy.

Reviewer #1: No

Reviewer #2: No
